# A Rare Case of Appendicectomy in Situs Inversus: Challenges and Surgical Approaches

**DOI:** 10.7759/cureus.82289

**Published:** 2025-04-15

**Authors:** Nasrin Akter Jahan, Anup Banerjee, Alomgir Jalil Pramanik

**Affiliations:** 1 General Surgery, Evercare Specialized Hospital, Rangpur, BGD; 2 Acute Internal Medicine, Stepping Hill Hospital, Manchester, GBR; 3 General Surgery, Rangpur Medical College Hospital, Rangpur, BGD

**Keywords:** laparoscopic appendectomy, left lower quadrant pain, left-sided acute appendicitis, situs inversus totalis, situs inversus with dextrocardia

## Abstract

Left-sided acute appendicitis (LSAA) is an extremely rare cause of acute abdominal pain, and even the most experienced surgeons may encounter it only a few times in their careers. While acute appendicitis remains the most common indication for emergency gastrointestinal (GI) surgery, its diagnosis is typically straightforward. However, in cases of situs inversus totalis (SIT), the atypical anatomical positioning can obscure the clinical picture and lead to diagnostic uncertainty, often mimicking other left-sided pathologies and potentially resulting in delayed treatment and complications. This case report presents the surgical challenges encountered during an appendicectomy in a patient with SIT, emphasizing the importance of thorough preoperative imaging and meticulous intraoperative navigation. A nine-year-old boy with no prior history of SIT presented with left iliac fossa pain, fever, and vomiting - symptoms consistent with acute appendicitis. Clinical examination and diagnostic workup, including laboratory tests, abdominal ultrasound, and echocardiogram, confirmed an inflamed appendix located in the left lower quadrant with dextrocardia. Given the altered anatomical configuration, a laparoscopic appendicectomy was performed with particular attention to the reversed anatomy. The procedure was completed successfully, and the patient had an uneventful postoperative recovery. Although rare, documenting such cases in detail is essential to enhance clinical awareness and guide effective management. Increasing familiarity with LSAA in SIT can help reduce diagnostic delays and surgical complications while contributing to the broader understanding of rare anatomical variants in surgical practice.

## Introduction

Situs inversus totalis (SIT) is a rare congenital condition characterized by a complete mirror-image reversal of the thoracic and abdominal organs. Its incidence ranges from 0.001% to 0.01% in the general population [1,2]. In patients with SIT, the appendix is located in the left lower quadrant (LLQ), which can lead to left-sided acute appendicitis (LSAA). The incidence of acute appendicitis in individuals with SIT is estimated to be between 0.016% and 0.020% [3].

Diagnosing LSAA poses a clinical challenge due to its atypical presentation. While acute appendicitis typically presents with right lower quadrant (RLQ) abdominal pain, LSAA may mimic other causes of LLQ pain, such as acute diverticulitis, sigmoid volvulus, ureteric colic, colonic carcinoma, and gynecological conditions, including ectopic pregnancy and ovarian torsion [4,5]. Additionally, about 14.7% of SIT patients may still present with RLQ pain despite left-sided inflammation due to atypical neural pathways, further complicating the clinical picture [4]. Imaging is critical in such cases. While ultrasonography is often the first-line imaging modality, its utility is limited in certain anatomical variants or in obese patients. In contrast, contrast-enhanced computed tomography (CECT) offers superior diagnostic accuracy and can simultaneously identify reversed anatomy and inflamed appendiceal tissue [4,6]. Radiological clues, such as dextrocardia on chest X-ray or a right-sided gastric bubble on abdominal films, may alert clinicians to the possibility of SIT [7]. Delays in the diagnosis of LSAA can result in increased rates of complications, including perforation, peritonitis, and intra-abdominal abscess formation [4,6]. Therefore, a high index of suspicion, especially in patients presenting with atypical abdominal pain, is crucial. Management of LSAA in SIT is generally consistent with standard appendicitis treatment protocols. Laparoscopic appendectomy is preferred due to its diagnostic and therapeutic advantages, including better visualization of intra-abdominal anatomy and reduced postoperative complications [2,5,6]. However, it requires awareness of the reversed anatomy and adjustment in port placement and instrument handling [2,6].

We present a rare and instructive case of laparoscopic appendectomy in a patient with SIT, highlighting the diagnostic challenges, intraoperative complexities, and the necessity for a tailored surgical approach to effectively manage this uncommon clinical scenario.

## Case presentation

A nine-year-old boy with no known history of situs inversus presented to the hospital with dull abdominal pain that had persisted for one day. The patient reported that the pain initially began in the periumbilical region and gradually migrated to the left iliac fossa, where it became sharper and more intense over time. Associated symptoms included nausea and febrile shivering.

On examination, the patient was febrile with a temperature of 101.4 °F, tachycardic, and hemodynamically stable. Abdominal examination revealed a soft abdomen with minimal tenderness and rebound tenderness elicited in the left iliac fossa (corresponding to McBurney’s point on the left). Chest auscultation was unremarkable; however, the apex beat was auscultated in the right fifth intercostal space with no audible murmurs.

Laboratory investigations revealed a total leukocyte count of 15.8 × 10⁹/L with neutrophilic predominance (8.4 × 10⁹/L) (Table 1). Liver function tests (LFTs), urea and electrolytes (U&E), and renal function tests (RFTs) were all within normal limits.

**Table 1:** Laboratory results of the patient on admission showing the full blood count

Parameter	Patient value	Normal Value
WCC (White cell count)	15.8× 10⁹/L	(3.7-11.0× 10⁹/L)
Hb (Hemoglobulin)	135 g/L	(115-165) g/L
Neutrophils	8.4× 10⁹/L	(1.7-7.5× 10⁹/L)

**Figure 1:**
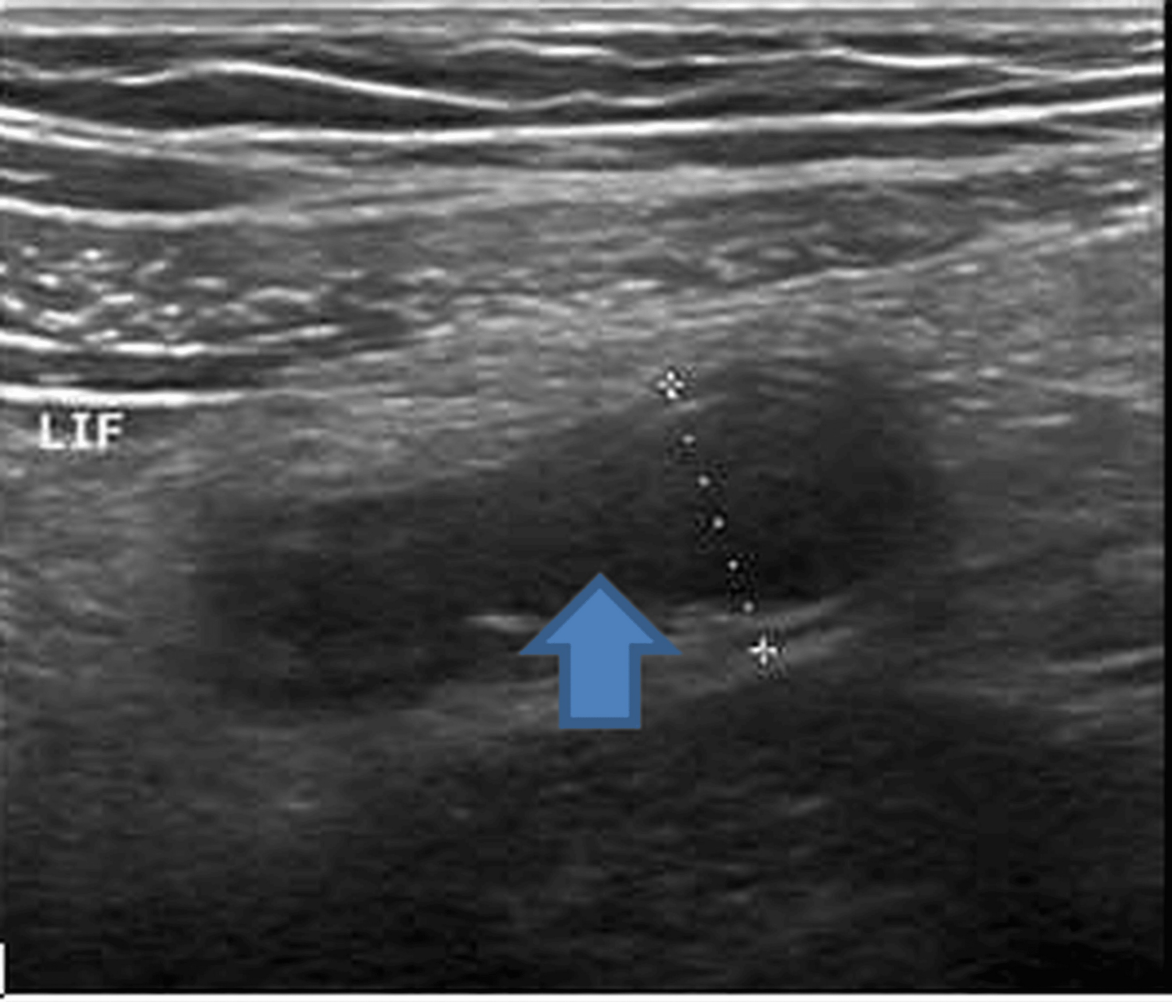
US abdomen of the patient showing dilated tubular structure in left iliac fossa suggestive of acute appendicitis

Abdominal ultrasonography showed probe tenderness in the left iliac fossa and provided evidence of situs inversus: the liver was visualized in the left hypochondrium and the spleen in the right hypochondrium, both with normal echogenicity and echotexture, features suggestive of left-sided acute appendicitis; a dilated (11 mm in diameter), blind-ended tubular structure was noted in the left iliac fossa with a slightly thickened, hypoechoic wall and echogenic peri-appendiceal fat. No free fluid was observed in the peritoneal cavity (Figure 1).

**Figure 2:**
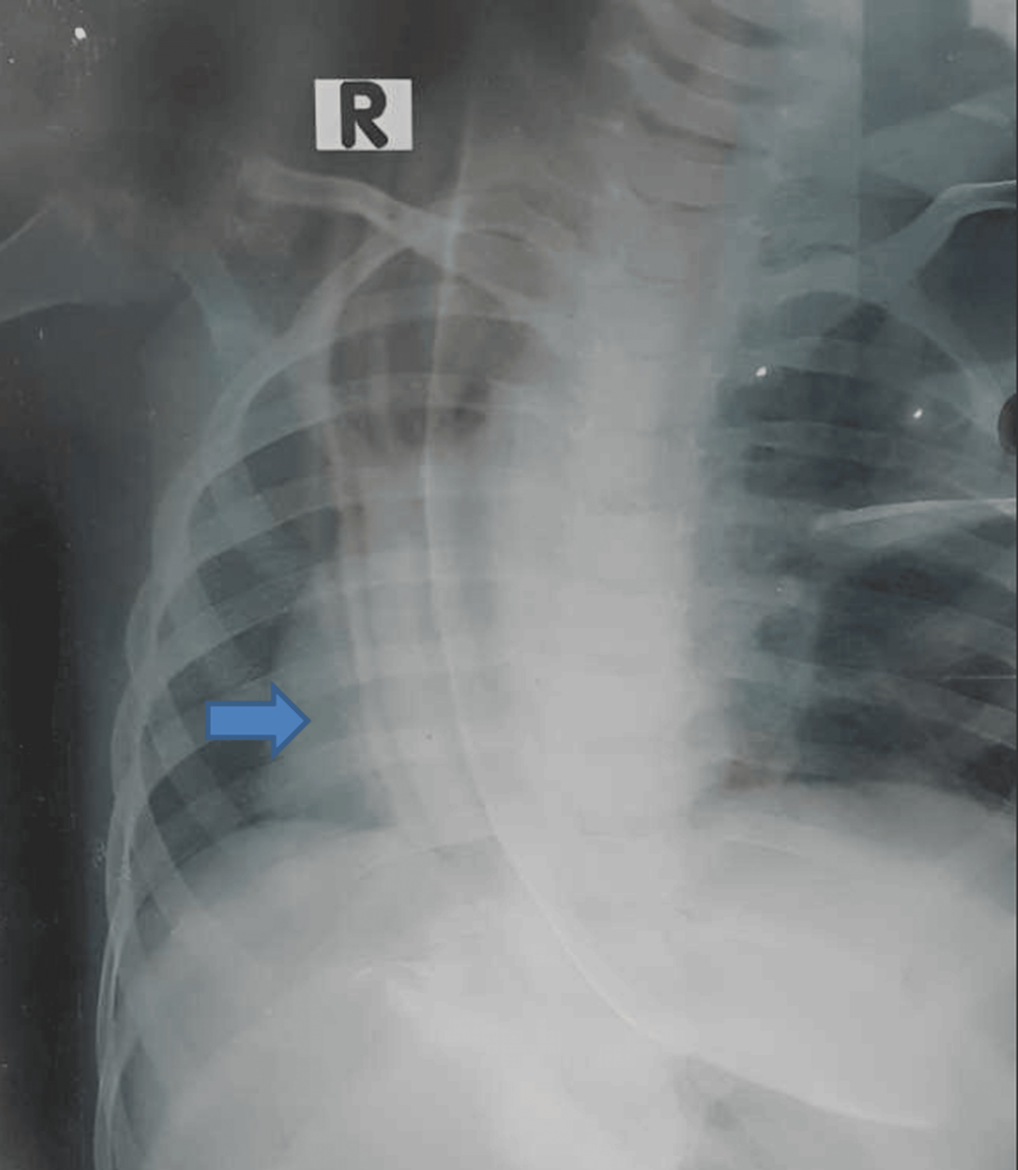
Chest X-ray of the patient showing dextrocardia

A chest radiograph revealed a cardiac shadow on the right side (Figure 2).

An echocardiogram with color Doppler confirmed mirror-image dextrocardia and provided the impression of situs solitus with mirror-image dextrocardia (Table 2).

**Table 2:** Echocardiogram report of the patient showing mirror image dextrocardia TR: tricuspid regurgitation; PASP: pulmonary artery systolic pressure

Test	Result
Echocardiogram	Situs solitus; Mirror image dextrocardia
	Trivial TR with calculated PASP: 26 mmHg
	Good biventricular function
	No pericardial effusion

Based on the findings, diagnostic laparoscopy and appendectomy were scheduled, with informed consent obtained in accordance with the gold standard of care.

Surgical procedure

Port placement was determined by the surgical team’s experience, ensuring optimal ergonomics for the procedure. A 10 mm trocar was inserted just below the umbilicus, with two additional 5 mm trocars placed in the left iliac fossa and suprapubic region. The lead surgeon and first assistant operated from the patient’s right side, while the second assistant was positioned on the left. A video monitor was placed near the patient’s feet on the left side for optimal visualization.

Intraoperative findings were consistent with the preoperative imaging. The liver and gallbladder were located on the left side, while the greater curvature of the stomach was observed on the right. A thorough examination of the entire abdominal cavity was performed, with all solid organs appearing normal. The inflamed appendix was located and successfully removed. The patient recovered well postoperatively and was discharged two days later without complications.

## Discussion

Situs inversus usually does not cause any adverse health issues by itself, and many individuals with the condition lead normal, healthy lives. It is often detected incidentally during imaging studies, such as X-rays, CT scans, or ultrasounds, performed for unrelated medical issues. In some cases, situs inversus may be associated with other conditions, such as Kartagener syndrome, which involves chronic respiratory infections, bronchiectasis, and sinusitis [1].

The human body exhibits three types of visceral situs: situs solitus, situs inversus, and situs ambiguous [4]. Situs solitus refers to the normal anatomical arrangement of thoracic and abdominal organs. Situs inversus is characterized by a mirror-image arrangement of these organs, whereas situs inversus totalis is a rare congenital condition resulting from autosomal recessive inheritance, where all thoracoabdominal organs are completely reversed [5].

In patients with situs inversus, the altered anatomical configuration can make the diagnosis of acute conditions more challenging. In emergencies, this may result in delayed diagnosis and management, as the clinical signs are not typical and may be easily misinterpreted [6]. Clinicians must maintain a high index of suspicion for atypical presentations of appendicitis, particularly in individuals with known congenital anomalies or abnormal cardiac positioning such as dextrocardia [4].

The differential diagnosis for left lower quadrant pain includes diverticulitis, renal colic, ruptured ovarian cyst, Meckel’s diverticulitis, epididymitis, strangulated hernia, bowel obstruction, psoas abscess, and appendicitis, which, in rare anatomical variations, may also present on the left side [2].

The diagnosis of LSAA in patients with situs inversus is based on a combination of clinical examination, imaging, and surgical exploration. Important diagnostic tools include abdominal ultrasound, CT scan, chest X-ray (to detect dextrocardia), diagnostic laparoscopy, and relevant blood tests [1]. Imaging plays a critical role in identifying appendicitis in the context of reversed anatomy [2].

Laparoscopic appendectomy is the preferred surgical approach in these cases, offering a minimally invasive option with excellent visualization of the abdominal cavity [2]. However, surgeons must be prepared to adapt their technique to accommodate the mirror-image anatomy, which may affect port placement, hand dominance, and instrument handling. Patients should also be informed of their unique anatomical condition to aid in the management of any future medical or surgical interventions.

## Conclusions

A patient presenting with left lower abdominal pain and dextrocardia on a chest radiograph should raise suspicion for acute appendicitis associated with situs inversus totalis (SIT). Preoperative imaging is essential for confirming the diagnosis and aiding in effective surgical planning. Despite the anatomical challenges posed by reversed organ positioning, laparoscopic appendectomy remains the preferred surgical approach due to its minimally invasive nature, enhanced visualization, and adaptability. While the surgical technique may vary depending on the surgeon’s preference and comfort, a tailored approach ensures both safety and efficacy in managing this rare clinical presentation.
